# Work-life conflict and musculoskeletal disorders: a cross-sectional study of an unexplored association

**DOI:** 10.1186/1471-2474-12-60

**Published:** 2011-03-16

**Authors:** Oliver Hämmig, Michaela Knecht, Thomas Läubli, Georg F Bauer

**Affiliations:** 1 Division of Public and Organizational Health, Institute of Social and Preventive Medicine, University of Zurich, Hirschengraben 84, 8001 Zurich, Switzerland; 2 Center for Organizational and Occupational Sciences, Swiss Federal Institute of Technology Zurich (ETH Zurich), Kreuzplatz 5, 8032 Zurich, Switzerland

## Abstract

**Background:**

The health consequences of work-family or rather work-life conflict (WLC) have been studied by numerous researchers. The work-related causes of musculoskeletal disorders (MSD) are also well explored. And stress (at work) has been found to be a consequence of WLC as well as a cause of MSD. But very little is known about a potential association between WLC and MSD and the possible mediating role of stress in this relationship.

**Methods:**

Survey data collected in 2007 among the workforces of four large companies in Switzerland were used for this study. The study population covered 6091 employees. As the exposure variable and hypothesized risk factor for MSD, WLC was measured by using a 10-item scale based on an established 18-item scale on work-family conflict. The outcome variables used as indicators of MSD were (low) back pain and neck/shoulder pain. Stress as the assumed intervening variable was assessed by a validated single-item measure of general stress perception. Correlation coefficients (r), standardized regression coefficients (β) and multiple adjusted odds ratios (OR) were calculated as measures of association.

**Results:**

WLC was found to be quite strongly associated with MSD (β = .21). This association turned out to be substantially confounded by physical strain at work, workload and job autonomy and was considerably reduced but far from being completely eliminated after adjusting for general stress as another identified risk factor of MSD and a proven strong correlate of WLC (r = .44). A significant and relevant association still remained (β = .10) after having controlled for all considered covariates. This association could be fully attributed to only one direction of WLC, namely the work-to-life conflict. In subsequent analyses, a clear gradient between this WLC direction and both types of MSD was found, and proved to be consistent for both men and women. Employees who were most exposed to such work-to-life conflict were also most at risk and showed a fivefold higher prevalence rate (19%-42%) and also an up to sixfold increased relative risk (OR = 3.8-6.3) of suffering greatly from these types of MSD compared with the least exposed reference group showing very low WLC in this direction. Including stress in the regression models again reduced the strength of the association significantly (OR = 1.9-4.1), giving an indication for a possible indirect effect of WLC on MSD mediated by stress.

**Conclusion:**

Future research and workplace interventions for the prevention of MSD need to consider WLC as an important stressor, and the MSD risk factor identified in this study.

## Background

### Research on musculoskeletal disorders

There is a long tradition of research into the causes of musculoskeletal disorders (MSD) in occupational medicine. There is also substantial and consistent evidence that MSD are strongly work-related [1]. Moreover, MSD are a major source of disability and lost work time [2]. It has been shown repeatedly that MSD are a predominant cause of sick leave and absence from work, particularly among blue-collar workers [3,4]. MSD represent the most prevalent work-related health problem in Europe and the main occupational disease suffered by European workers, accounting for over 50% of all occupational diseases in the EU [5] and for 40% of the work-related health costs worldwide [4].

The work-related causes, or rather risk factors of MSD, have been well explored [1,6-10]. There is clear evidence that MSD are directly caused by strenuous working conditions such as lifting and carrying heavy loads, poor posture, tiring positions, vibrations or highly repetitive movements [1,9,11,12]. Besides physical or rather biomechanical risk factors, specific psychosocial work demands and occupational strains were studied in relation to MSD. In particular, work-related psychosocial factors such as time pressure or rather a fast pace of work, monotonous tasks, low job control, low job satisfaction, lack of social support at work, high workload or even work overload in terms of volume, and perceived job stress as well as psychosocial distress in general have been identified and recognised as predictors or risk factors for MSD [1,8,11-18]. There is consensus among researchers that MSD related to physical aspects at work and strenuous working conditions are on the decline, while those related to stress, excessive work demands, and other psychosocial work factors are on the increase [5].

In contrast to the research effort that has been made in recent years on psychosocial factors at work in relation to MSD, very few or not any studies at all focused on psychosocial aspects outside the work environment. In particular, incompatible demands and role conflicts between job and family have been completely neglected in MSD research.

### Research on work-family/life conflict

In recent years, just as much attention has been paid to the problem of reconciling work and family life and its antecedents and consequences in occupational health psychology as to MSD and their causes in occupational medicine. Under the headword of work-family conflict (WFC), a lot of research has been done over the last two decades on negative spillover and inter-role conflicts between the two life domains, showing that such conflicts and spillover effects are strongly associated with various health problems, and in particular with psychosocial ill health. In more specific terms, WFC has been found and identified as a risk factor for mental health in general and as a stressor or predictor of psychological distress in particular [19-30]. Health-related outcomes found in addition to stress were increased substance abuse, more frequent depressions and mental disorders, occurrence of burnout syndrome and various psychosomatic stress symptoms including lack of appetite, sleep disorders, headache and fatigue.

Recently, individual studies on this subject matter use the more inclusive and comprehensive term of work-life conflict (WLC) in order to overcome the traditionally narrow focus on role conflicts between work and family and to document the broader scope and concept and expanded study population by including employees without their own core families or children living at home [19]. The present study also takes this approach.

Studies on WFC or WLC focus mainly on well-being and mental health outcomes, psychosomatic symptoms or adverse health behaviours, largely ignoring other important public and occupational health issues and problems such as social inequalities in health, physical inactivity, and also MSD.

### A "blind spot" in both fields of research

We have pointed out that MSD research so far suffers from a lack of examining WLC as a possible risk factor for musculoskeletal health, and research into WFC and WLC has so far largely ignored MSD as a potential result of role conflicts at the work-home interface. Whereas stress has been recognised as a risk factor for MSD, it has also been studied and identified as an outcome of WLC. However, there has been no contact between these two research fields in the past. At least until recently, when the European Foundation for the Improvement of Living and Working Conditions reported a certain association between work-life balance and MSD for the first time [5]. But this association has not been studied in detail, nor indeed adjusted for any control or confounding variables, and was based on data from the European Working Conditions Survey, which are greatly limited as regards the measurement of WLC. Apart from this report on 'Managing musculoskeletal disorders' and our own study [19], which provides initial scientific evidence and reference for an association between WLC and backache and therefore justifies the present continuative and in-depth study, we are unaware of any study that has been carried out and published on the assumed relationship between WLC and MSD.

### Study aims and hypotheses

Stress is assumed to be the link in this potential but neglected and unexplored relationship. WLC as the identified stressor is expected to lead to stress symptoms and subsequently to physiological stress responses that are considered to cause MSD, particularly upper extremity disorders such as neck and shoulder pain [31]. In brief, it is proposed that perceived stress in response to WLC contributes to MSD. In addition, WLC may hinder active recovery of the stressed musculoskeletal system due to physical inactivity as a result of missed time and/or physical or emotional exhaustion. It has been shown that active recovery plays an important role in predicting individual health [31], and that individual well-being and musculoskeletal health in particular benefit from leisure activities in general and from physical activities and leisure-time exercise in particular [32,33]. Moreover, WLC presumably also hinders passive recovery and relaxation due to sleeping problems or insomnia that have been identified as its mental health outcomes [19].

**Figure 1:**
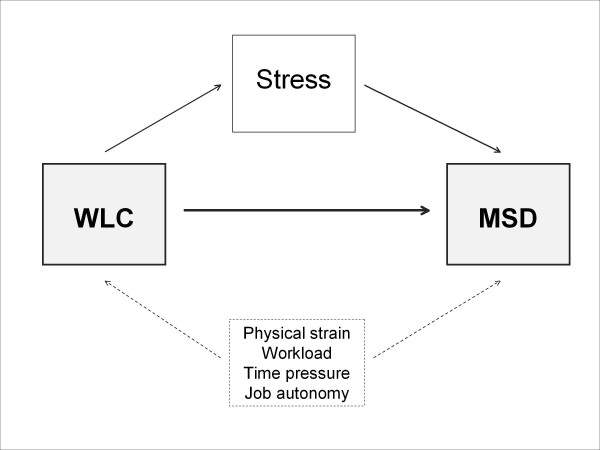
**Conceptual path model**.

Against this background and in the light of such theoretical considerations, the main aim of the present study was to fill this existing research gap by investigating the potential but so far unexplored association between WLC as the main risk factor and MSD as the outcome under study. In doing so, the study also aimed to estimate or rather eliminate any potential confounding and in particular to consider and adjust for stress as the supposed link or intervening variable in this association. More precisely, the study aimed to find initial supportive evidence for the following hypotheses or postulated causal pathways illustrated in the conceptual path model (see Figure 1).

1. WLC as the identified stressor is assumed to be a psychosocial risk factor for musculoskeletal health and is therefore expected to be significantly and strongly associated with MSD (causational hypothesis).

2. Specific working conditions such as physical strain at work, time pressure at work, workload and job autonomy are recognised or at least supposed correlates of both WLC and MSD and may therefore confound the relationship (confounding hypothesis).

3. The association between WLC and MSD is suspected to be partly or even completely mediated by stress, i.e. the inclusion of stress as another independent variable is expected to reduce or even eliminate the association between WLC and MSD (mediational hypothesis).

## Methods

### Data collection and study sample

The data used for this study were collected cross-sectionally in 2007 by postal and online surveys using a fully standardised and comprehensive questionnaire and mostly well-established and validated multiple- and single-item measures of exposure (work-life conflict) and outcome (stress feelings, back or low back pain, neck or shoulder pain). The employee survey was conducted among the workforces of four large and well-known companies from different industries (insurance, banking, transportation, and healthcare) of the service sector in German-speaking Switzerland, or rather in the area of greater Zurich. The participating companies were (1) Swiss Re (survey at their Zurich headquarters), one of the world's leading reinsurance companies, (2) Zurich Cantonal Bank (company-wide survey), the third largest Swiss bank after UBS and Credit Suisse, (3) Swissport International (survey on its site at Zurich airport), a global cargo and aircraft ground services company, and (4) the Cantonal Hospital of Winterthur (company-wide survey), a large state hospital in the canton of Zurich. A total of 6,091 employees were surveyed, distributed as follows among the companies, or rather industries: insurance (n = 1,696), banking (n = 3,127), transportation (n = 766), healthcare (n = 502).

Data were collected online in the two participating companies which had computers and an intranet available to the whole workforce (banking, insurance), whereas the other two companies which were without full computer and intranet access (healthcare, transportation) carried out a postal survey with a paper support questionnaire. In one company (healthcare), a stratified random sample of the personnel was taken. In all the other companies, full samples were used. Nearly 11'000 employees were asked to complete the questionnaire and allowed to do this during their working time or at home at their request, and return it anonymously. The overall return or response rate of the employees selected for the survey was 56%.

The study is observational and not clinical or experimental, did not involve drugs, and the data were collected on a voluntary and anonymous basis, and in particular not from hospitals, retirement homes or prisons. This meant that no approval was required by the ethics committee nor any authorisation by the commissioner for data protection by the national and cantonal laws nor were these recommended by the medical-ethical guidelines for scientific integrity of the Central Ethics Committee and the Swiss Academies of Sciences.

The consolidated study population covers employees across both sexes and all ages, educational levels, job positions, and many occupational categories. However, it is not a complete cross section of the entire employed population in German-speaking Switzerland. Compared to a subsample of the Swiss Household Panel (SHP), which is representative for the whole employed population in Switzerland, the study population shows a significant over-representation of men, young and middle-aged people between 30 and 40 years of age, well-educated persons, and Swiss citizens as well as those in higher occupational positions and full-time jobs (see Table 1). In contrast, the study population shows an under-representation of female, older, unskilled and foreign employees as well as those of low job status and in part-time employment.

**Table 1:** Study population in comparison with a nationally representative sample of employees of the Swiss Household Panel (SHP)

		Study population	**Standard population**^1^)
		Survey 2007 (N = 6091)	SHP 2007 (N = 3885)
Sex	Men	**57.1%**	49.0%
	Women	**42.9%**	51.0%

Age	(15)-30 years	25.0%	28.4%
	31-40 years	**31.9%**	23.0%
	41-50 years	27.4%	26.0%
	51 years and older	**15.8%**	22.5%

Education(highest level achieved)	No vocational education	**5.8%**	19.1%
	Basic vocational education (apprenticeship)	37.1%	39.2%
	University-entrance diploma (high-school graduation)	6.5%	9.8%
	Higher vocational education	**30.8%**	16.0%
	University	19.7%	15.9%

Nationality	Swiss (incl. dual citizenship)	**87.6%**	78.8%
	Other nationality	**12.4%**	21.2%

Number of persons living in household	1 (mostly singles)	18.9%	19.8%
	2 (mostly couples or single parents with one child)	**36.9%**	29.2%
	3+ (mostly families with children and/or other relatives)	**44.2%**	51.0%

Family status	No underage children living in own household	60.2%	61.4%
	At least one underage child living in own household	39.8%	38.6%

Job status	Management position (member of managing board)	5.2%	4.7%
	Supervisory/training position	**34.7%**	24.1%
	Production position (standard level)	**60.1%**	71.2%

Employment status	Part-time (< 20 h/week)	**5.3%**	17.4%
	Part-time (20-39 h/week)	21.4%	24.2%
	Full-time (40+ h/week)	**73.3%**	58.4%

Bold figures = proportions of subpopulations of the study sample differing significantly from the standard population

^1) ^A representative and weighted sample of employees of private companies or government organisations of working age between 15 and 65 years and with permanent residence in Switzerland (with employees of private households or partners in a relative's firm being excluded)

### Measures

#### Work-life conflict

The construct of WFC has been conceptualised and recognised as multidimensional, and in particular as bidirectional [34-37], by considering three forms (time-, strain- and behaviour-based conflict) and two directions (work-to-family and family-to-work conflict). Researchers have measured WFC in many ways, still largely focusing on the time-and strain-based forms and only one direction, namely on work conflicting or interfering with the family (and not vice versa). In the present study, the same forms of conflict were considered, but from both directions.

As a measure of the more inclusive WLC, we used an adapted and shortened version of the well-established and validated 18-item WFC scale of Carlson et al. [34]. The most suitable items were selected from the original scale, translated into German and assessed on the 5-point Likert scale with response categories ranging from 'completely agree' (score 4) to 'completely disagree' (score 0). The term 'family' was then replaced by expressions comprising the whole non-work domain. The resulting 10-item measure used in this study covers four of the six recognised dimensions of the multidimensional construct of WFC, or rather WLC, namely strain-based work-to-life conflict (e.g. 'When I come home from work, I am often too tired to take part in family or private activities'), time-based work-to-life conflict (e.g. 'I regularly miss private events or family activities because of my work'), strain-based life-to-work conflict (e.g. 'Due to stress and obligations in my personal life, I often find it very difficult to concentrate at work') and time-based life-to-work conflict (e.g. 'My family and personal obligations often keep me from participating in work events which are important for my career'). The items assessing the behavioural-based forms, which are difficult to understand conceptually as well as linguistically, especially in their German translation, were not adopted or used in the questionnaire.

A principal component analysis with varimax rotation resulted in two factors with an eigenvalue greater than 1 explaining 60.6% of the variance and indicating the two causal directions of the construct, namely the work-to-life conflict and the life-to-work conflict. The two forms of each direction, the strain-based and the time-based form, could not be reproduced by the principal component analysis.

We then created an accumulated and consolidated 10-item scale with values ranging from 0 (no conflict) to 40 (very strong conflict), and alternatively two 5-item subscales with sum scores between 0 and 20 that indicate the two directions of the construct. A reliability analysis resulted in good Cronbach's alpha coefficients as a measure of internal consistency for the consolidated 10-item scale (α = .81) as well as for the two subscales measuring the work-to-life conflict (α = .86) and the life-to-work conflict (α = .80).

#### General stress

Psychological stress was assessed by a general indicator of stress symptoms developed in the early 1970 s, a single-item measure that has been validated by Elo et al. [38]. This single item refers to the general experience of stress and not specifically to work-related stress. First, the following definition of stress was given: "Stress means a situation in which a person feels tense, restless, nervous and anxious and/or is unable to sleep at night because his/her mind is troubled all the time." Second, after this definition the question "How often have you felt stressed in the last 12 months?" was asked. The response was recorded on a 4-point scale varying from 'Never' (score 1) to 'Very often' (4). For reasons of compatibility and comparability with other Swiss studies and data sources, the question and response were slightly modified compared to the original wording and the number of response categories [38].

#### Musculosceletal disorders

MSD were assessed by the question 'Have you had any of the following pains or medical complaints in the last 4 weeks?' (Yes, serious/Yes, a little/No, not at all) and the following two items: (1) Back pain or pain in the lower back; (2) Neck ache or shoulder pain. These complaints are most commonly reported by employees. Upper limb disorders, another main group of MSD commonly known as 'repetitive strain injuries', were not covered.

For a consolidated statistical analysis, a summary score ranging from 0 to 4 was created by adding up the values of the two 3-point scaled MSD items (with scores of 0 'No, not at all', 1 'Yes, a little' and 2 'Yes, serious'). For a differentiated statistical analysis, the two items were dichotomized and recoded as binary-coded dummy variables (with a score of 0 for no or low pain and a score of 1 for considerable pain) in order to perform separate multiple-logistic regression analyses.

#### Confounding variables

The only variables that are available in the data set and are expected to be correlated with both the exposure and outcome variables are the following: time pressure at work, workload, physical strain at work and job autonomy. These work-related factors - three risk factors and one protective factor - have been proven to increase or decrease job stress and the risk of MSD.

Single-item measures were used to assess the three work-related risk factors for MSD that could also affect WLC. The corresponding questions ('I suffer from constant time pressure due to a heavy workload', 'Over the past year, my job has become more and more demanding', 'My work is physically strenuous') were taken from the effort-reward imbalance questionnaire of Siegrist et al. [39], each with a 'Yes/No' response combined with an indication of stress level in the case of an affirmative answer. Job autonomy as a protective factor was assessed by a multiple-item measure, a selection of six items about how and when the job gets done. The questions included in the questionnaire relate to having some influence on the amount of work to be done and especially to the freedom to decide if and when to take a break without permission, to take single days off at short notice or vacations (response scale ranging from 0 'Never' to 4 'Always'). The 6-item measure consists of five items taken from two well-validated multiple-item scales on influence and degree of freedom at work of the Copenhagen Psychosocial Questionnaire of Kristensen et al. [40], supplemented by our own formulated item ('Can you take some days off at short notice?'). Cronbach's alpha of the 6-item scale was .76.

### Statistical analysis

To test our basic assumptions as pictured in the conceptual path model (see Figure 1), we first analysed the bivariate associations between the independent or exposure variable (WLC), the dependent or outcome variable (MSD), the supposed intervening variable (stress) and the assumed confounding variables, and estimated several Pearson's correlation coefficients (r). After that, multiple linear regression analyses were performed and standardized regression or rather beta coefficients (β) were calculated in order to explore and identify WLC and perceived general stress as risk factors or possible predictors of MSD as well as to investigate confounding in this relationship. For this reason, four regression models were calculated stepwise. We initially controlled only for the commonly used control variables such as age, sex, education and additionally for physical activity as a proven protective factor for MSD. We then made additional adjustments for the potential work-related confounding variables (physical strain, workload, time pressure, job autonomy). Subsequently, we added the general stress indicator as another important potential covariate to the fully adjusted regression model in order to estimate the direct and independent effect of WLC on MSD (see Figure 1). And finally, to investigate possible differentiated effects of the two directions of the WLC construct, the consolidated 10-item measure was replaced by the two separated 5-item subscales.

As a next step, we counted the cases (absolute frequencies) and computed the percentages (relative frequencies) by levels of exposure to the stressor, i.e. by degree of WLC, or rather by degree of the previously identified more relevant direction of WLC, and we did this for both measured types of MSD separately (backache or low back pain, neck or shoulder pain) and stratified by sex.

Finally, for a more detailed and differentiated analysis, we computed two multivariate logistic regression models with multiple adjusted odds ratios (OR) as proxies for the relative risk, and did so for each of the two measured types of MSD as the dichotomous outcomes and for both sexes separately. We initially started with a *total effect model *with the selected direction of WLC as the exclusive risk factor (besides the covariates or confounding variables respectively). We then contrasted this with a *direct effect model *using stress as an additional risk factor that presumably mediates the association between WLC and MSD and consequently reduces or eliminates the total effect and reveals the residual direct effect of WLC on MSD.

## Results

The simple correlation matrix in Table 2 revealed highly significant intercorrelations between all relevant study variables without exception, providing evidence for an unadjusted association between WLC and MSD, substantiating the suspicion of confounding in this association, supporting the assumption of WLC as a strong stressor and indicating a mediational relationship. There was a highly significant bivariate correlation between the independent or exposure variable (WLC) and the dependent or outcome variable (MSD) as expected (r = .20). In addition, the work-related variables of physical strain at work, workload, time pressure at work and job autonomy correlated consistently with both consolidated multiple-item measures of WLC and MSD and therefore had to be considered as potential confounders in a further multivariate analysis of association, or rather regression. Also, the assumed intervening variable of general stress was strongly correlated with both the dependent (r = .23) and independent (r = .44) variables.

**Table 2:** Intercorrelations between all relevant study variables (Pearson's r)

		Score	M	SD	1	2	3	4	5	6	7
1	Musculoskeletal disorders	0-4	1.28	1.11	-						
2	Work-life conflict	0-40	10.47	5.59	.20***	-					
3	Physical strain	0-4	0.67	1.00	.16***	.18***	**-**				
4	Workload	0-4	1.73	1.18	.13***	.31***	.30***	**-**			
5	Time pressure	0-4	1.95	1.05	.11***	.35***	.26***	.57***	**-**		
6	Job autonomy	0-24	14.15	4.61	-.19***	-.25***	-.39***	-.18***	-.25***	**-**	
7	General stress	1-4	2.20	0.76	.23***	.44***	.09***	.26***	.30***	-.13***	-

*p≤.05; **p < .01; ***p < .001; n.s.= not significant (p > .05); bold print = correlation coefficients of special focus for the study

With these preconditions satisfied, only one last condition remained to meet all sufficient statistical criteria for mediation according to Baron and Kenny's three-step approach for testing mediation with cross-sectional data [41]: the effect of the independent variable on the dependent variable must be less when the intervening variable is controlled. To study this and the other hypotheses (see Figure 1), four multiple linear regression models were computed using either the consolidated WLC measure or the two differentiated WLC subscales as the independent or exposure variable(s), and the MSD summary score as the dependent or outcome variable (see Table 3). The effect of WLC on MSD was initially adjusted for the control variables (Model 1), then additionally adjusted for the confounding variables (Model 2), subsequently also controlled for the intervening variable (Model 3) and finally calculated separately using the two differentiated WLC subscales instead of the consolidated measure (Model 4). Overall, we found clear evidence for an association and confounding as well as support and indication for mediation if possible with cross-sectional data.

**Table 3:** Work-life conflict and stress as the main explanatory factors and other covariates of musculoskeletal disorders

Dependent variable	Model 1	Model 2	Model 3	Model 4
			
Musculosketal disorders (Score 0-4)	*β*	*β*	*β*	***β***
*Explanatory/intervening variable(s):*				
Work-life conflict consolidated (0-40)	.21***	.16***	.10***	-
Work-life conflict differentiated				
Life-to-work conflict (0-20)	-	-	-	**.01**
Work-to-life conflict (0-20)	-	-	-	.12***
General stress (1-4)	-	-	.17***	.17***
*Confounding variables:*				
Physical strain at work (0-4)	-	.08***	.08***	.08***
Workload (0-4)	-	.05**	.04*	.03
Time pressure at work (0-4)	-	-.01	-.03	-.04*
Job autonomy (0-24)	-	-.06***	-.06***	-.05**
*Control variables:*				
Age	.04**	.02	.04**	.03*
Sex (male)	-.15***	-.13***	-.12***	-.12***
Education (highest level achieved: 0-10)	-.10***	-.07***	-.07***	-.08***
Physical activity (0-7)	-.04***	-.05***	-.04***	-.04**

Adjusted R-Squared	.079	.095	.115	.118

Number of cases in model	5641	5431	5426	5425

*p≤.05; **p < .01; ***p < .001; bold print = beta coefficients of special focus for the study

These multiple linear regression analyses (see Table 3), stepwise including and adjusting for additional covariates, showed that the association initially found (β = .21) and controlled for the usual control variables (Model 1) was considerably reduced when firstly an adjustment was made for the considered work-related confounders (Model 2), and secondly, the intervening stress variable as the assumed mediator was additionally added to the regression model (Model 3). The reduction of effect size due to these adjustments was substantial. Overall, the beta coefficient as the measure of association halved, and the stress variable finally turned out to be the stronger risk factor for MSD (β = .17) than WLC. However, there still remained a significant and relevant association between WLC and MSD (β = .10), as shown in the fully adjusted regression model (Model 3). The completely specified and differentiated regression model (Model 4) came out as the best fit of all four computed linear regression models, and explained the highest percentage of the total variance (adjusted R squared = 11.8%). This model showed the multiple adjusted effects of the two assessed and considered directions of WLC separately and identified work-to-life conflict as the only predictive and MSD-relevant direction of WLC. The other measured direction of WLC, namely life-to-work conflict, turned out to be not at all predictive with regard to MSD. In other words, the association found between WLC and MSD can be ascribed exclusively to the effect of the work-to-life conflict. The two WLC directions or subscales were not that strongly correlated with each other (r = .24), so that their combined use in a regression analysis was not problematic with regard to likely collinearity.

Stratified analyses now focusing solely on work-to-life conflict as the only remaining explanatory variable basically confirmed the association between WLC and MSD for both measured types of MSD and for both sexes separately.

Table 4 shows the number of cases (absolute frequencies) and the percentages (relative frequencies) for both measured types of MSD and both sexes separately, each differentiated by degree of exposure (work-to-life conflict). The results revealed that percentages of backache or low back pain as well as of neck or shoulder pain increase greatly and gradually with an increasing degree of work-to-life conflict, especially in women. Percentages in the most exposed group (very high work-to-life conflict) are five times higher throughout than in the least exposed group (very low work-to-life conflict), independent of sex or type of MSD.

**Table 4:** Number of cases and percentages of strong (low) back pain and neck/shoulder pain in men and women by degree of work-to-life conflict

	Suffer a lot from backache or lower back pain				Suffer a lot from neck or shoulder pain			
		
	Men		Women		Men		Women	
				
	N	%	N	%	N	%	N	%
*Work-to-life conflict*								
Very low (0-3)	23	4.2	25	5.6	28	5.1	37	8.2
Low (4-7)	97	7.4	76	8.9	79	6.0	117	13.6
Moderate (8-11)	86	9.1	80	11.0	97	10.3	149	20.3
High (12-15)	53	12.1	69	19.1	69	15.9	109	30.1
Very high (16-20)	18	19.1	24	27.3	23	24.2	37	42.0
**Total**	**277**	**8.3**	**274**	**11.0**	**296**	**8.9**	**449**	**18.0**

Multivariate logistic regression analyses with multiple adjusted OR as measures of association also revealed that the relative risk of suffering seriously from backache or low back pain and neck or shoulder pain was highest in those who were most exposed to work-to-life conflict compared to the reference group comprising those least exposed, irrespective of type of MSD or sex (see Table 5). Multiple adjusted OR for this most exposed group ranged from 3.8 to 6.3 in the total effect model and from 1.9 to 4.1 in the direct effect model, depending on type of MSD and sex. A strong association and a clear gradient was found for both types of MSD and both sexes (total effect model). Even when perceived general stress was considered, i.e. when the corresponding intervening variable was included in the logistic regression model, adjusted OR were clearly reduced but still increased steadily with cumulative degree of work-to-life conflict (direct effect model). This applies basically to both types of MSD, although for back pain the increased OR mostly were no longer significant on the 95%-confidence interval level for the more exposed group (very high work-to-life conflict) compared to the reference group (very low work-to-life conflict). Again, as expected and already implied by the linear regression models (see Table 3), stress was found to reduce the strength of association and therefore suspected to mediate the relationship between WLC and MSD more (backache or low back pain) or less (neck or shoulder pain). Stress turned out to have quite a strong and independent effect on back and neck or shoulder pain and therefore to be a strong risk factor for MSD itself. Although the results of these stratified and differentiated logistic regression analyses were largely consistent with the findings of the previous and consolidated linear regression analyses, slightly differing results were found with regard to sex and type of MSD. Associations were stronger and gradients were more pronounced in women and for neck/shoulder pain, whereas stress-adjusted direct effects were smaller for (low) back pain.

**Table 5:** Work-to-life conflict as the principal risk factor for strong (low) back pain and neck/shoulder pain, stratified by sex and additionally adjusted for general stress

	Suffer a lot from backache or lower back pain				Suffer a lot from neck or shoulder pain			
		
	Men		Women		Men		Women	
				
	OR^†^	95%-CI	OR^†^	95%-CI	OR^†^	95%-CI	OR^†^	95%-CI
**Total effect model**	n = 3200		n = 2297		n = 3196		n = 2313	
*Work-to-life conflict*								
Very low (0-3)	1		1		1		1	
Low (4-7)	1.77	1.10-2.86	1.46	0.89-2.40	1.24	0.78-1.97	1.61	1.07-2.43
Moderate (8-11)	1.93	1.16-3.21	1.77	1.06-2.94	**1.99**	1.24-3.22	**2.49**	1.64-3.77
High (12-15)	**2.47**	1.40-4.36	**2.94**	1.70-5.11	**2.96**	1.74-5.06	**3.91**	2.47-6.19
Very high (16-20)	**3.77**	1.78-7.97	**4.12**	2.01-8.44	**4.82**	2.39-9.70	**6.30**	3.41-11.66

**Direct effect model**	n = 3197		n = 2296		n = 3193		n = 2311	
*Work-to-life conflict*								
Very low (0-3)	1		1		1		1	
Low (4-7)	1.65	1.01-2.67	1.21	0.73-2.00	1.11	0.69-1.78	1.45	0.96-2.19
Moderate (8-11)	1.62	0.97-2.72	1.29	0.77-2.17	1.64	1.00-2.67	**2.06**	1.35-3.14
High (12-15)	1.75	0.97-3.16	1.73	0.97-3.07	**2.19**	1.32-3.81	**2.83**	1.76-4.56
Very high (16-20)	2.28	1.04-4.99	1.86	0.87-3.96	**3.18**	1.53-6.60	**4.14**	2.18-7.87
*General stress*								
Never	1		1		1		1	
Sometimes	1.35	0.84-2.18	2.31	1.22-4.40	1.79	1.06-3.04	1.56	1.01-2.40
Often	**2.27**	1.33-3.87	**5.10**	2.59-10.02	**2.63**	1.48-4.67	**2.93**	1.83-4.69
Very often	**3.55**	1.90-6.62	**8.27**	4.00-17.13	**3.52**	1.82-6.84	**2.68**	1.55-4.62

^†^Controlled for age, education, physical activity and adjusted for potential work-related confounders such as physical strain at work, workload, time pressure at work and job autonomy; bold figures = highly significant OR (p < .01)

## Discussion

On the basis of cross-sectional data from a large-scale employee survey in the service sector with an extraordinary large and heterogeneous study sample of 6091 interviewed employees, we showed that WLC is significantly and quite strongly associated with MSD even when adjusting for various control variables (sex, age, education, physical activity) and other covariates (physical strain at work, time pressure at work, workload, job autonomy, general stress). In sum, we found more or less strong associations for all studied relationships and assumed causal paths, namely between WLC and MSD, between WLC and stress and between stress and MSD, and also for both measured types of MSD and both sexes separately. In addition, these associations showed clear gradients almost throughout, thus indicating possible dose-response relationships. The stress variable emerged as a strong correlate of WLC and an important and significant risk factor of MSD, obviously explaining at least part of the strong association initially found between WLC and MSD. As implied by the conceptual path model and in support of the three underlying study hypotheses to be tested, evidence was found for an association between WLC and MSD and for substantial confounding in this relationship as well as an indication for an indirect effect of WLC on MSD mediated by stress, even though the hypothesized causation and assumed mediation could not have been studied appropriately with cross-sectional data.

These results basically cannot be compared to the findings from other studies, simply because no other studies have so far examined a potential association between WLC and MSD. But apart from our main finding and research focus, our 'collateral' results confirm the findings of other cross-sectional and longitudinal studies on the positive association between work-related or general stress and neck and/or shoulder pain as found by Andersen et al. [12] and reviewed and reported by Larsson et al. [11], Punnett & Wegman [1] and Bongers et al. [17]. At the same time, our finding that general psychological stress is a strong risk factor for MSD itself and particularly for backache and low back pain disagrees with the conclusion of Hartvigsen et al. [16] who found only insufficient evidence for a positive association between stress at work and low back pain in their systematic review of prospective cohort studies. All in all, our study results add to the now extensive but still incomplete research and the partly insufficient or inconsistent evidence that psychosocial factors at work - but not at the work-home interface - increase the risk of MSD [1,8,11-13,15-18]. Furthermore, our findings are partly in line with the results of a recently published cross-sectional study of Saastamoinen et al. [42], who found, at least in women, both directions of WFC to be associated with acute and chronic general pain even though they did not look at musculoskeletal pain in particular.

### Strengths and limitations

The study has its strengths and its weaknesses. First of all and most important, it fills a research gap by investigating the association between WLC and MSD, a relevant topic that used to be a blind spot in both established fields of research for a long time and still remains under-studied. Second, the study sample used is impressively large, exceptionally heterogeneous, and in particular not limited to certain sub-populations or specific professions, thus basically allowing better statistical power and a broader scope to be achieved than are usual in WLC studies. Third, the use of a comprehensive multiple-item scale as the main exposure or explanatory variable - a shortened and adapted version of the validated 18-item WFC scale of Carlson et al. [34] - ensures a better measurement validity than in previous studies carried out in Switzerland [19,20] and therefore reduces the risk of a misclassification bias.

The criticism usually passed on cross-sectional study design, single-source survey data or poor measures in WFC research [43] can also be applied to the present study.

Due to its cross-sectional design, causality and mediation cannot be verified and reverse or reciprocal causation between WLC and MSD and particularly between WLC and stress or stress and MSD cannot be excluded either. Mediation is a hypothesized causal chain in which an antecedent variable affects a mediator variable that, in turn, affects a dependent variable. Cross-sectional data and analyses have been and still are widely used to test for mediation and to calculate such a mediated or indirect effect [44-46]. Baron and Kenny [41] proposed a three-step approach for testing mediation with cross-sectional data. We took the same approach by conducting several regression analyses. However, this most widely used statistical procedure or test based on cross-sectional data is limited in the usual way by the inability to draw inferences about causation and hence mediation, and by the possibility of producing biased estimates [45,47]. In other words, by using cross-sectional data for this study, we were limited in testing the conceptual path model (see Figure 1) and could in principle only test for association and not for causation or mediation. However, our findings at least produce a fairly good indication of causality in compliance with Hill's criteria of causation [48] as well as meeting all the required conditions for mediation according to Baron and Kenny [41]. Although the strong associations and clear gradients consistently found in the relationships studied and for the two types of MSD and both sexes, even after adjusting for all considered confounders and control variables and reducing this association significantly by adjusting for stress, do not prove the causational and mediational hypotheses, they do support them.

Due to the use of single-source self-reported survey data, a systematic bias is also basically possible as a result of common method variance. In this case, however, the conceptual distance between a psychosocial construct such as WLC and a cluster of clinical and physical health complaints makes a causal attribution of MSD to WLC by respondents and therefore a 'common method bias' not very plausible, the more so as such an association has so far been unexplored and unidentified.

The use of a single item as a global measure of general psychological (dis-)stress in the present study may also be criticised against the background of the ongoing debate on the validity of single-item measures compared to multiple-item measures and in view of an increasing use of multiple-item scales to measure complex multidimensional constructs in health and social sciences [19]. However, the single-item measure used for general stress perception has been satisfactorily validated by Elo et al. [38] and can substitute longer measurement scales without any particular concern.

A further shortcoming can be criticised. The non-random sampling of the companies participating in the survey produces a potential selection bias that can be estimated. The study sample is clearly not a cross section of the employed population in Switzerland and differs from a nationally representative sample of employees with regard to various socio-demographic characteristics such as sex, age, education, job status, employment status, marital status and nationality, just to mention a few. As highly educated employees as well as employees in high job positions and of Swiss nationality are over-represented, the study sample implies a common and frequently observed middle-class bias and refers mostly to white-collar employees who are by definition well-educated members of the middle class and ethnic majority, self-employed or employed by large organisations. However, unlike in most other studies in the field of research, this study population includes blue-collar workers or more precisely poorly qualified or unskilled employees and members of lower classes and/or ethnic minorities, even though they are under-represented.

A low participation or response rate may also result in a selection bias. The response rates of three of the four companies ranged between 52% and 68%, each of their samples being sufficiently representative of their workforces. The outlier in this regard with the lowest response rate by far (35%) was the fourth company, the operator of cargo and aircraft ground services. The high proportion of non-responders in this company was mainly caused by the exceptionally low response rate of the aircraft and baggage handling personnel (18%) as a result of unexpectedly heavy snowfall at Zurich airport during the data collection period. This greatly increased the workload for a short time and consequently reduced the willingness of staff to participate in the survey as reported by the person in charge of employee surveys. Since such selection, or rather 'self exclusion', from participating in the survey is not expected to be systematic or non-random, a selection bias as a consequence of this snowfall is implausible and not highly evident.

## Conclusions

The present study found convincing evidence for a largely unexplored association. WLC has been shown to be a major but so far unrecognised risk factor for MSD. Whereas MSD were found to be strongly work-related, and research has focused widely on the various working conditions and psychosocial work factors that increase their risk, little attention has been given to psychosocial aspects outside the direct work environment, and particularly to role conflicts and incompatible demands at the interface of paid work and private life. Therefore, future research on MSD needs to be more aware of the WLC issue and to consider WLC, and especially work-to-life conflict, as the important and strong stressor and likely risk factor for MSD that was recognised in this study. There is a particular need for additional cross-sectional and subsequent longitudinal studies to provide evidence in support of our study results and particularly of our study hypotheses.

Our results not only suggest the need for further research on the subject matter, but that - if replicated and supported by subsequent studies - may also have practical implications. In the past, workplace interventions for the promotion or protection of musculoskeletal health in general and the prevention of MSD in particular tended to focus on physical activity or on traditional ergonomics and human engineering. Now that initial evidence for this newly detected psychosocial risk factor has been provided, and once it is supported by subsequent studies, there may be a need for workplace or labour market interventions that help to avoid or minimize role conflicts between work and private life and/or to prevent such problems of reconcilability from resulting in general psychological stress that in turn seems to impair musculoskeletal health as suggested by the present study.

## Competing interests

The authors declare that they have no competing interests.

## Authors' contributions

OH conducted the study, prepared and revised the manuscript and performed the statistical analyses. MK was involved in constructing the questionnaire and collecting the data. MK, TL and GB contributed to the conception and design of the study and took an active part in the discussion and interpretation of the findings. All co-authors critically reviewed the manuscript and approved the version to be published.

## Pre-publication history

The pre-publication history for this paper can be accessed here:

http://www.biomedcentral.com/1471-2474/12/60/prepub
